# Subacute cutaneous lupus erythematosus following osimertinib therapy for non–small cell lung cancer: A case report

**DOI:** 10.1016/j.jdcr.2024.08.015

**Published:** 2024-08-31

**Authors:** Eun Jae Kim, Mia S. DeSimone, Connie R. Shi

**Affiliations:** a Harvard Medical School, Boston, Massachusetts; b Department of Pathology, Brigham and Women’s Hospital, Boston, Massachusetts; c Center for Cutaneous Oncology, Dana-Farber Cancer Institute, Boston, Massachusetts; d Department of Dermatology, Brigham and Women’s Hospital, Boston, Massachusetts

**Keywords:** case report, cutaneous adverse reaction, non–small cell lung cancer, osimertinib, subacute cutaneous lupus erythematosus

## Introduction

Epidermal growth factor receptor (EGFR) tyrosine kinase inhibitors (TKIs) are used in the treatment of non–small cell lung cancer (NSCLC) with *EGFR* mutations.^1^ Osimertinib is a third-generation EGFR TKI approved for treatment of NSCLC with common EGFR mutations. Common dermatologic adverse events of osimertinib include pruritus, acneiform eruption, xerosis, and paronychia, among other rashes.^2^ Here, we present a case of subacute cutaneous lupus erythematosus (SCLE) arising in a patient with NSCLC treated with osimertinib.

## Case report

**Fig 1:**
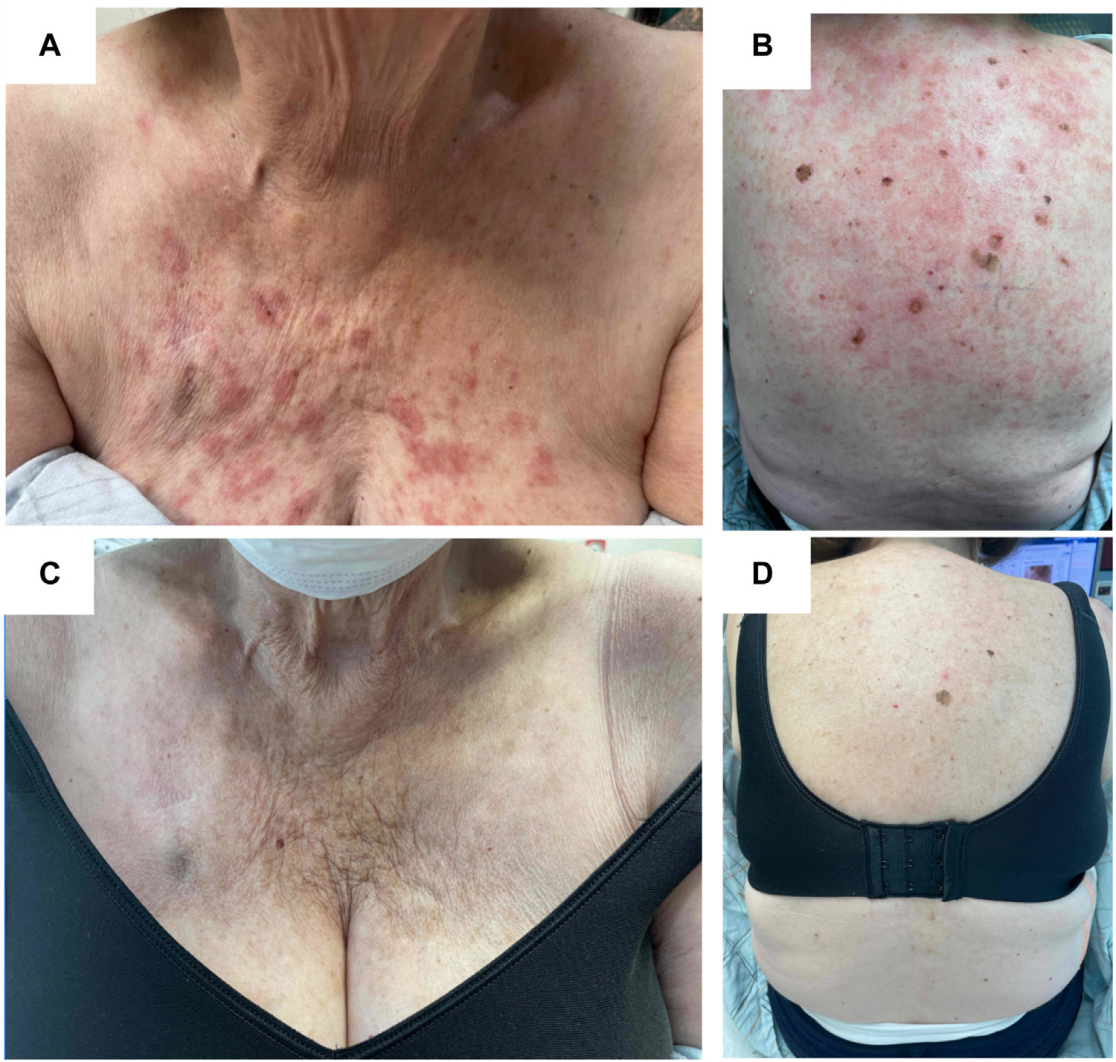
Drug-induced subacute cutaneous lupus erythematosus after osimertinib therapy. **A, B,** Pink papules and thin plaques on the chest and back in photodistributed areas at 2 months after starting osimertinib therapy. **C, D,** Significant improvement of cutaneous lesions was seen at 4 months after osimertinib dose reduction and treatment with topical corticosteroid ointment.

**Fig 2:**
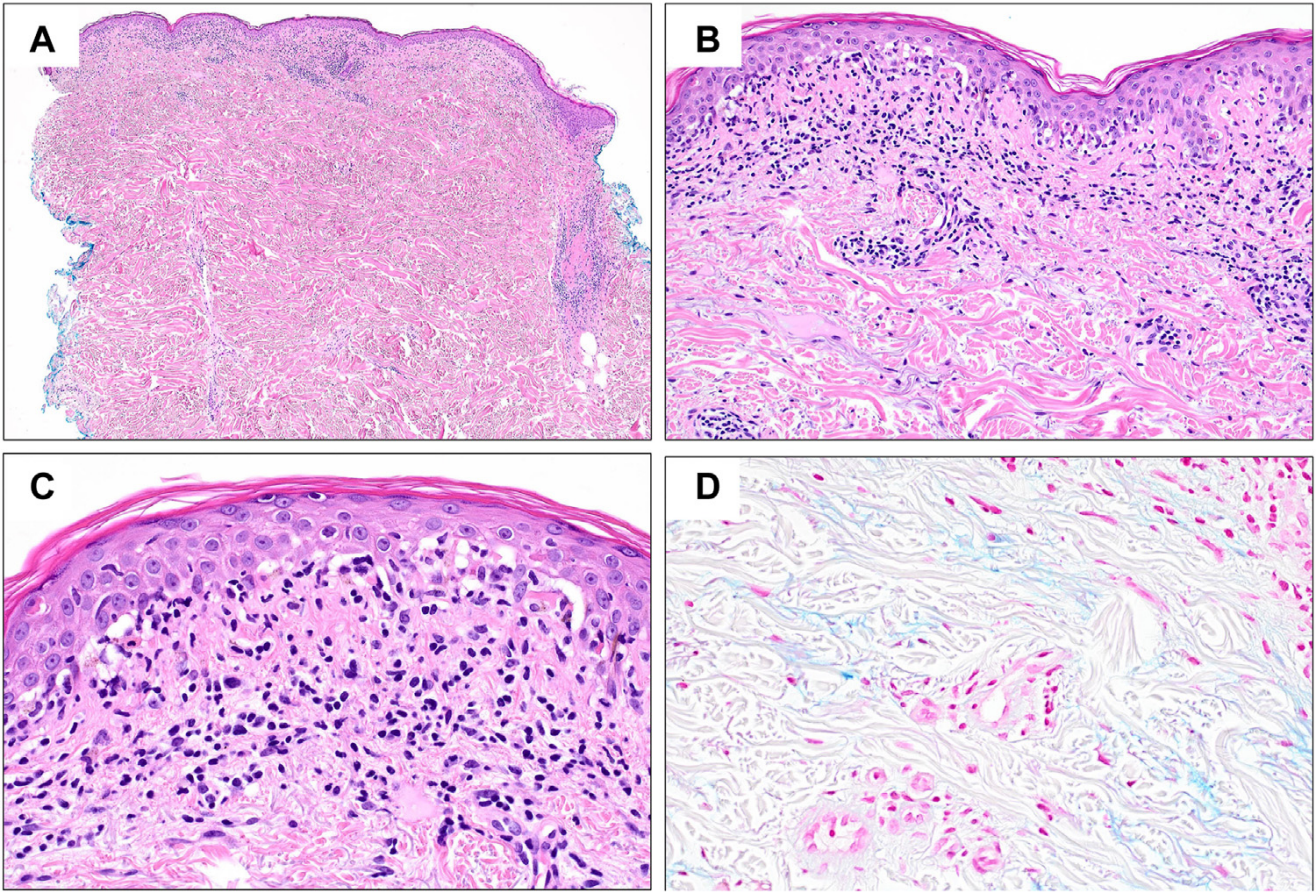
Histopathology of skin punch biopsy from the patient’s back. **A,** The sections show epidermal atrophy with papillary and reticular perivascular and periadnexal inflammatory infiltrate. **B, C,** At medium and high magnification, there is vacuolar interface dermatitis with dyskeratotic keratinocytes, cytoid bodies, and focal satellite cell necrosis. **D,** An Alcian blue stain highlights increased interstitial mucin in the dermis. (**A-C,** Hematoxylin-eosin stain; original magnifications: **A,** ×40; **B,** ×200; **C** and **D,** ×400.)

An 81-year-old woman initiated osimertinib for treatment of EGFR-mutated stage III NSCLC. After 2 months receiving osimertinib at 80 mg daily, she experienced mildly pruritic, scaly, pink papules, and thin plaques in photodistributed areas on the chest, back, and upper extremities (Fig 1, *A* and *B*). She reported no other associated symptoms including fever, myalgias, or joint pain. A punch biopsy from the back revealed vacuolar interface dermatitis with superficial and deep perivascular and periadnexal predominantly lymphocytic infiltrate (Fig 2, *A-C*). An Alcian blue stain highlighted increased dermal mucin deposition (Fig 2, *D*). The histopathologic findings were consistent with connective tissue disease. Laboratory studies revealed positive antinuclear antibodies at 1:320. Anti-Ro (SS-A) antibodies were elevated (>8.0), and anti-La (SS-B) and antidouble stranded DNA antibodies were both negative. Her renal function was within normal limits: serum creatinine level was 0.92 mg/dL, and urinalysis showed no proteinuria. Based on laboratory and clinical findings, a diagnosis of SCLE was rendered.

Given the patient’s advanced age and absence of personal or family history of autoimmune disease, drug-induced SCLE was suspected. Aside from osimertinib, her medication history included aspirin (>10 years), amlodipine (>10 years), simvastatin (>10 years), lisinopril (>10 years), and labetalol (1 month, previously taking metoprolol, and labetalol was later reintroduced after rash resolution with no recurrence after 3 months of rechallenge). The patient was not taking proton pump inhibitors or any over-the-counter medications or supplements.

Osimertinib was identified as the most likely culprit of the drug-induced SCLE. Because of the importance of continuing osimertinib for treatment of her NSCLC, the decision was made in conjunction with her oncologist to continue osimertinib while managing the rash supportively. Subsequently, her osimertinib dose was decreased to 40 mg daily because of hyponatremia; all other medication dosages were maintained. Her rash was managed with 0.05% topical betamethasone ointment twice daily to the affected areas on the trunk and extremities. Systemic therapy with hydroxychloroquine was also discussed but deferred by the patient. There was progressive improvement with topical therapy alone, and the patient eventually achieved near-complete clearance of her lesions at 4 months (Fig 1, *C* and *D*) while continuing reduced-dose osimertinib and achieving continued response in her NSCLC.

## Discussion

SCLE is a subtype of cutaneous lupus erythematosus that is characterized by photosensitive psoriasiform or annular lesions that typically involve sun-exposed sites. Drug-induced SCLE shares clinical, histopathologic, and immunologic features with idiopathic SCLE.^3^ A wide range of medication classes have been implicated in drug-induced SCLE, most commonly diuretics (eg, hydrochlorothiazide), antifungals (eg, terbinafine), proton pump inhibitors, chemotherapeutics, and biologics.^4^^,^^5^ The culprit drug can be challenging to identify in drug-induced SCLE given the variability in the timing of disease onset in relation to drug exposure. Disease resolution typically occurs within weeks of culprit drug cessation.^5^ Symptoms can be managed with topical therapy such as topical corticosteroids and calcineurin inhibitors, or with systemic treatment using antimalarials.^3^ Our patient fortunately experienced improvement in the rash with topical therapy and did not require cessation of any of her medications including osimertinib, thereby allowing her to continue this important treatment for her NSCLC.

In our patient, SCLE likely relates to osimertinib therapy given the timing of initiation relative to rash onset. Although she did have longstanding exposures to other medications (ie, lisinopril and simvastatin) that have been previously implicated in cases of drug-induced SCLE, the patient had been taking these medications for over a decade before SCLE onset. She did not have any other new medication exposure except for labetalol, which has not previously been associated with drug-induced SCLE, only cases of drug-induced systemic lupus erythematosus.^6^ Furthermore, she had previously tolerated a different β-blocker without cutaneous side effects and tolerated resumption of labetalol after SCLE resolution with no rash recurrence after 3 months. Using the Naranjo adverse drug reaction probability scale,^7^ osimertinib scored the highest likelihood of being associated with this eruption (6; probable), whereas her other longstanding chronic medications scored either 1 or 2 (possible). Although it is not possible to definitively exclude labetalol and lisinopril as causative agents of our patient’s eruption, they were much less likely than osimertinib in this case.

Two prior reports have linked EGFR TKIs to lupus erythematosus-like eruptions, among which there has only been one case of SCLE.^8^^,^^9^ After treatment with erlotinib, a first-generation EGFR TKI, 1 patient experienced fever, erythematous patches, and butterfly-shaped erythema over the cheeks consistent with a systemic lupus erythematosus-like reaction.^8^ In the second case, a patient developed drug-induced SCLE with erlotinib therapy, then experienced exacerbation of cutaneous lesions after switching to osimertinib and was treated with systemic steroids to manage the eruption.^9^ Although the exact pathogenesis of EGFR TKI-induced SCLE remains unclear, inhibition of EGFR has been shown to increase proinflammatory mediators in epithelial cells.^10^ The resultant cell apoptosis and inflammation lead to innate and adaptive immune activation that may contribute to the development of SCLE skin lesions.^9^^,^^11^ It is also possible that osimertinib-induced immune activation does not directly cause SCLE but rather unmasks the patient’s underlying genetic predisposition toward inflammatory dermatoses. Further research is needed to elucidate the precise pathogenic relationship between EGFR TKIs and SCLE.

For patients who experience SCLE related to EGFR TKI therapy, both this case and the previously reported case in the literature support continuation of EGFR TKI therapy with concomitant supportive care for the cutaneous eruption. In our case, the osimertinib dose was subsequently reduced because of hyponatremia, which may also have assisted in management of her rash. Unlike the previously reported case in the literature of SCLE related to EGFR TKI therapy,^9^ our case demonstrates that topical therapy alone can manage this cutaneous eruption without the need for systemic steroids or other systemic immunomodulatory agents and that targeted therapy can successfully be continued without systemic immunosuppression to treat drug-induced SCLE in this context. This case demonstrates successful continuation of EGFR TKI with drug-induced SCLE without systemic immunosuppression, which is of particular relevance given the importance of minimizing unnecessary immunosuppression and polypharmacy in oncology patients.

## Conclusion

Our case highlights the onset of SCLE after initiation of osimertinib in a patient with NSCLC. In this case, drug-induced SCLE was effectively managed on a lower osimertinib dose and supportive care with topical steroids and without the addition of systemic immunosuppression. Timely recognition and management of cutaneous adverse effects in patients undergoing treatment with osimertinib and other TKIs are essential to optimizing control of cutaneous symptoms while allowing patients to continue targeted cancer therapy.

## Conflicts of interest

None disclosed.
